# Economic Analysis of Mobile Integrated Health Care Delivered by Emergency Medical Services Paramedic Teams

**DOI:** 10.1001/jamanetworkopen.2021.0055

**Published:** 2021-02-24

**Authors:** Feng Xie, Jiajun Yan, Gina Agarwal, Richard Ferron

**Affiliations:** 1 Department of Health Research Methods, Evidence and Impact, McMaster University, Hamilton, Ontario, Canada; 2 Centre for Health Economics and Policy Analysis (CHEPA), McMaster University, Hamilton, Ontario, Canada; 3 Department of Family Medicine, McMaster University, Hamilton, Ontario, Canada; 4 Niagara Emergency Medical Services, Ontario, Canada

## Abstract

**Importance:**

Mobile integrated health care (MIH) is a new model of community-based health care to provide on-site urgent or nonurgent care. Niagara emergency medical services (NEMS) started MIH in 2018 to serve the Niagara region of Ontario, Canada. However, its economic impact is unknown.

**Objective:**

To compare time on task and cost between MIH and ambulance delivered by NEMS from a public payer’s perspective.

**Design, Setting, and Participants:**

This economic evaluation was an analysis of the NEMS databases regarding responses to emergency calls by the NEMS from 2016 to 2019. Emergency calls serviced by MIH in 2018 to 2019 were used as an intervention cohort. Propensity score matching was used to identify a 1:1 matched cohort of calls serviced by regular ambulance response for the same period and 2 years prior. Statistical analyses were performed from January to April 2020.

**Exposures:**

MIH compared with matched ambulance services.

**Main Outcomes and Measures:**

The main outcomes were the time on task (including time on scene and time at hospital) and costs. Costs were calculated in 2019 Canadian dollars using cost per minute and compared with the 3 ambulance cohorts.

**Results:**

In 2018 to 2019, there were 1740 calls serviced by MIH for which a matched ambulance cohort was identified for the same period and 2 years prior. The mean (SD) time on task was 72.7 (51.0) minutes for MIH, compared with 84.1 (52.0) minutes, 84.3 (54.1) minutes, and 79.4 (42.0) minutes for matched ambulance in 2018 to 2019, 2017 to 2018, and 2016 to 2017, respectively. Of calls serviced by MIH, 498 (28.6%) required ED transport (ie, after MIH team assessment, transport to ED was deemed to be necessary or demanded by the patient), compared with 1300 (74.7%) calls serviced by ambulance in 2018 to 2019, 1294 (74.4%) in 2017 to 2018, and 1359 (78.1%) in 2016 to 2017. The mean (SD) total cost per 1000 calls was $122 760 ($78 635) for MIH compared with $294 336 ($97 245), $299 797 ($104 456), and $297 269 ($81 144) for regular ambulance responses in the 3 matched cohorts, respectively.

**Conclusions and Relevance:**

Compared with regular ambulance response, MIH was associated with a substantial reduction in the proportion of patients transported to the ED, leading to a substantial saving in total costs. This finding suggests that the MIH model is a promising and viable solution to meeting urgent health care needs in the community, while substantially improving the use of scarce health care resources.

## Key Points

**Question:**

Is mobile integrated health care (MIH) delivered by emergency medical services more efficient than regular ambulance responses in addressing the needs of urgent care in the community?

**Findings:**

This economic evaluation compared 1740 calls serviced by MIH in 2018 to 2019 with propensity score–matched ambulance calls for the same period and 2 years prior and found that MIH was associated with a decrease in the proportion of patients transported to the emergency department and saved health care costs compared with regular ambulance responses.

**Meaning:**

These findings suggest that MIH is a promising and viable solution to meeting urgent health care needs while improving the efficiency in using emergency care resources.

## Teaser

This economic analysis examines whether mobile integrated health care delivered via emergency medical services (EMS) for emergency calls is associated with cost savings and reduced time on task compared with regular ambulance responses.

## Introduction

Health care systems are facing ever-increasing pressure to meet rising health care demand with limited resources. To address this issue, we must improve efficiency and reduce any wasteful spending in the system.

Mobile integrated health care (MIH) is a new model of community-based health care that uses community paramedics, who work directly with other health care professionals, to provide needs-based on-site urgent and nonurgent care.^1^ The concept of MIH is to address specific health care needs of patients in their homes or within mobile environments and provide innovative approaches to health care that traditionally require emergency medical services (EMS), emergency department (ED) care, or hospital admission. This model was initially designed as a proactive means to improve health care access for underserved rural populations. It has since been adapted to real-time reactive response to 911 calls as a safe, timely, and mobile medical care delivery model in the community setting.

MIH programs can improve quality of life, reduce ED transport and admission, and reduce hospitalization for frequent users of the ED.^2^ Existing MIH models have focused on the needs of specific community populations such as older adults with multiple chronic conditions,^3,4^ individuals at high risk,^5^ or individuals with acute mental health crises.^6^ These types of MIH programs are delivered with physician oversight by interprofessional teams or community paramedics. MIH, which is integrated into and delivered by trained paramedics in partnership with other health care professionals, could also provide an efficient care service and reduce unnecessary use of ED and hospital resources.^7^

Canadian literature^8^ shows that 23% of EMS calls are low urgency, at Canadian Triage and Acuity Scale level 4 or 5. These patients may have been cared for in other ways, potentially by primary care. Indeed, among those individuals calling EMS frequently, most experienced substantial loneliness and social isolation and had poor quality of life. Other responses to these EMS calls may be able to satisfy health care needs appropriately. In Canada, the MIH model has been incorporated into EMS in some jurisdictions. However, little is known about the performance of the MIH model delivered by EMS. The primary objective of this study was to compare the time on task and costs between MIH and matched ambulance services from a public health care payer’s perspective using real-world data routinely collected by the EMS system.

## Methods

This economic evaluation was an analysis of administrative databases without contact with any patient or individual. Therefore, neither an ethics review board’s approval nor informed consent was required, per 45 CFR §46. This study followed the relevant sections of the Consolidated Health Economic Evaluation Reporting Standards (CHEERS) reporting guideline.

### Niagara EMS MIH Program

Niagara EMS (NEMS) responds to unscheduled out-of-hospital emergent and nonemergent medical events for the Niagara Region of Ontario, Canada, a municipality of 1854 km^2^ with a 2016 census population of 447 888.^9^ NEMS operates an ambulance communication center with a land-based ambulance service that provides primary and advanced care paramedic services that approximate the scope of services provided by US emergency medical technicians and paramedics, respectively.^10^

Since July 1, 2018, NEMS has implemented an MIH program that was devised to provide innovative and efficient responses to low-urgency and nondifferentiated emergency calls, which have accounted for one-third of call volume since its creation. The MIH teams primarily provide unscheduled services to those meeting the criteria, except for some scheduled follow-up visits between calls. Table 1 provides a brief description of the program.

**Table 1.** Description of Mobile Integrated Health Care Implemented by Niagara Emergency Medical Services

Mobile integrated health team	Staffing	Focus
Community Assessment and Referral (CARE)	One advanced care paramedic, with community paramedic training	Low-urgency medical calls; providing patients with community resources to address identified medical and social needs
Falls Intervention Team (FIT)	One primary or advanced care paramedic with community paramedic training and one occupational therapist	Low-urgency falls, proactive visits to repeat callers for falls; providing in-home evaluation and education, links to select community resources to address identified medical and social needs^a^
Mental Health and Addictions Response Team (MHART)	One advanced care paramedic with community paramedic training and one mental health nurse	Calls with a chief concern of mental health and/or addictions issues, including overdose; providing patients with community resources, referrals for ongoing medical or social needs, harm reduction training and supplies^b^

^a^ This involves scheduled follow-up visits with patients who have had multiple falls to provide an assessment to seek ways to increase independence and therefore decrease reliance on emergency medical services.

^b^ This involves scheduled follow-up visits to known locations where drug use occurs and providing harm-reduction kits.

### Data Sources

The information for all emergency calls received and responded to from 2016 to 2019 was retrieved from the NEMS database, which records the location and priority description of the call, age and sex of the patient, the description of the response vehicle, whether transport to ED was required, and service times. Cost data were retrieved from the NEMS accounting database, including all equipment costs, operating costs, and salary and benefits of all staff employed.

### Propensity Score Matching

Propensity score matching is a widely used matching method that mimics a randomized controlled design from an observational study on the basis of particular characteristics.^11^ This method balances propensity scores between treated and untreated participants, achieving a similar distribution of baseline characteristics between the matched groups.

The MIH model was started in July 2018 with 4 teams deployed to supplement ambulance services. All calls serviced by the MIH team between July 1, 2018, and June 31, 2019, were used as an intervention cohort. Propensity score matching was used to identify a matched cohort of calls managed by EMS ambulance for the same period. Because EMS practices and budgets change over time, we also identified a matched cohort from each of the 2 years prior to the MIH implementation (2016-2017 and 2017-2018) to control for potential confounding of time. The variables used to identify the matching cohorts included age and sex of the patient, location, the time of year, and assigned call priority based on Medical Priority Dispatch System determinants. These measures describe the urgency and nature of the patient’s condition and scene circumstance and thus were included as a key variable to identify matched cohorts.

We applied and compared different propensity score matching methods. Nearest neighbor matching first selects a treated participant and then a matched participant whose propensity score is closest to that of the treated participant, while the optimal matching method matches participants so as to minimize the mean within-pair difference in propensity scores.^11^ Caliper nearest neighbor matching is a modified nearest neighbor matching that matches 2 participants only if the absolute difference in their propensity score is within a prespecified caliper distance.^11^ In our analyses, nearest neighbor matching within a caliper distance of 0.2 performed better than optimal matching. Therefore, for the base-case analysis, we chose nearest neighbor matching, with optimal matching included in the sensitivity analysis. The propensity score matching was conducted using R statistical software version 3.5.3 (R Project for Statistical Computing) and its MatchIt Package. Tests of statistical significance were run on all the variables used to drive the propensity scores, and there was no significant difference between the intervention and matched control cohorts.

### Time on Task

Two time intervals recorded in the EMS system are time on scene (ie, from response to either final disposition on scene if no patient transported or to arrival at hospital if there was transport) and time at hospital (ie, from arrival at hospital until transfer of care to ED, only applicable for those with an ED transport). The mean time on task was calculated as the sum of time on scene and time at hospital and was compared between the MIH and the matched ambulance cohorts. We also compared the mean time on task for those with and without ED transport separately.

### Costs Associated With MIH and Ambulance Services

Canada has a single-payer, publicly funded health care system. Therefore, our economic analysis was conducted from the public payer’s perspective. Costs incurred for the provision of health care, whether by EMS or hospital, are all relevant and included in the analysis.

Three types of costs associated with the MIH and ambulance services were included in the analysis, namely, capital cost, operating cost, and personnel cost. Capital cost included the expenses on vehicles and equipment. The annual vehicle cost was calculated by amortizing the total cost of a vehicle over 54 months as per the standard practice in EMS. An ambulance was equipped with a monitor, a stretcher and batteries, a stair chair and enhancement, a tablet, a drug-dispensing machine, and a laptop with preinstalled software. An MIH vehicle was equipped with a monitor, a tablet, a drug-dispensing machine, and a laptop with the same software. The cost of any software license was equally distributed among all ambulance and MIH vehicles. Operating cost included expenses for fuel, maintenance, and repair of the service vehicle.

There are 2 types of personnel in NEMS: on-vehicle staff and administration staff. We used the annual salary and benefits to estimate the personnel cost. On-vehicle staffs include advanced care paramedics, primary care paramedics, registered nurses, and occupational therapists. Because both MIH and ambulances were operating in 2018 to 2019, we used the ratio of the total service time by MIH to that by ambulance to allocate the administration personnel costs between the 2 models for this period in the base case analysis. All costs were adjusted to 2019 Canadian dollars using the inflation rates published by Bank of Canada (1 CAD$ = $0.7537 US in 2019).

### Base-Case Cost Analysis

We converted the time on task to cost and compared the total cost per 1000 calls associated with the MIH model vs the ambulance service. First, we calculated per minute cost by dividing cost by time in minutes. It is necessary that we differentiated between operating cost and capital or personnel cost in calculating the mean cost per minute. The latter is the cost incurred regardless of whether a vehicle is in service (ie, fixed costs), whereas the former is directly determined by the time in service (ie, variable costs). Therefore, mean operating cost per minute was calculated by dividing the cost by total service time of the year for all the vehicles of same type (Equation 1).


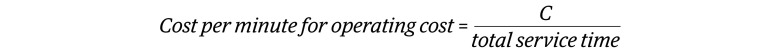


Mean capital and personnel costs per minute were calculated by dividing the cost (*C*) by total vehicle-time of the year (Equation 2).


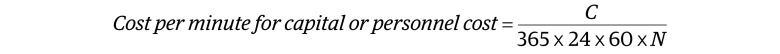


Where *C* denotes capital cost or personnel cost and *N* the number of MIH vehicles or ambulance. The 3 types of cost per minute were summed to derive the mean cost per minute.

Second, we calculated the mean cost per call for on-scene task by multiplying the mean cost per minute with the mean time on scene, and the mean cost per call for hospital transport by multiplying the cost with the mean time at hospital. Each of the mean cost per call was multiplied by 1000 to derive the total cost. In addition, the cost of an ED visit was calculated by multiplying $184 (estimated cost per ED visit in Ontario adjusted to 2019 Canadian dollars)^12^ by the number of ED transports. The total cost associated with each service model was, therefore, calculated using Equation 3:


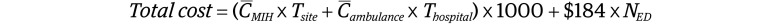


where *C̅* denotes the mean cost per minute, *T* the mean time on task, and *N_ED_* the number of ED transports per 1000 calls. It is important to note that for ambulance transport, the same mean cost per call was used for both on-scene task and hospital transport. For MIH, the mean cost per call for on-scene task was the cost derived for MIH, but the mean cost per call for hospital transport was the cost derived for ambulance transport of the same year because an ambulance was always dispatched and transferred the patient to ED even for those calls initially served by the MIH team.

### Statistical Analysis

In the sensitivity analyses, we used propensity optimal matching to identify the matched cohorts. For the costing, we used the ratio of the number of MIH vehicles to the number of ambulances to allocate the administration cost between the MIH and ambulance services for 2018 to 2019. Furthermore, we excluded the administration personnel cost when calculating the cost per minute in the sensitivity analysis in a scenario assuming that the budget for administration was fixed and would not be changed even with the operation of the MIH program.

Statistical tests were conducted using *t* test for continuous variables and χ^2^ test for categorical variables. A 2-tailed *P* < .05 was considered significant. Statistical analyses were performed using R statistical software version 3.5.3 (R Project for Statistical Computing) from January to April 2020.

## Results

### Description of the Emergency Calls

There were 56 652 emergency calls received by the NEMS between July 1, 2018, and June 30, 2019, compared with 52 536 calls in 2016 to 2017 and 55 523 calls in 2017 to 2018. People needing EMS services were more likely to be male and younger in 2018 to 2019 compared with the previous 2 years (eTable 1 in the Supplement). There were also substantial differences in terms of monthly volume, location, and priority of the calls between the years (eTable 2 in the Supplement).

### Propensity Score Matching

There were 1743 emergency calls serviced by the MIH team between July 1, 2018, and June 30, 2019. Except for 3 MIH calls, the propensity score–matching algorithm successfully identified 1740 matched calls for the same period as well as for 2017 to 2018. There were 1739 matched calls identified for 2016 to 2017.

### Mean Times on Scene and on Hospital Transport

As shown in Table 2, of the 1740 calls, 498 (28.6%) serviced by MIH had ED transport (ie, after MIH team assessment transport to ED was deemed to be necessary or demanded by the patient), compared with 1300 (74.7%) serviced by ambulance in 2018 to 2019, 1294 (74.4%) in 2017 to 2018, and 1359 (78.1%) in 2016 to 2017. The MIH service was associated with a mean (SD) of 60.97 (26.28) minutes on scene and 61.02 (53.44) minutes at the hospital for these calls with ED transport. The corresponding mean (SD) times on scene and at the hospital for regular ambulance response were 41.08 (14.59) minutes and 56.12 (48.88) minutes in 2018-2019, 40.42 (12.53) minutes and 58.01 (51.91) minutes in 2017-2018, and 40.31 (13.09) minutes and 49.38 (36.98) minutes for 2016-2017. For those without ED transport, the MIH spent a mean (SD) of 53.00 (27.20) minutes on scene, compared with 45.44 (17.91) minutes for 2018 to 2019, 43.62 (17.77) minutes for 2017 to 2018, and 42.77 (19.72) minutes for 2016 to 2017 (Table 2).

**Table 2.** Times on Task by Mobile Integrated Health Care and Regular Ambulance Response

Characteristic	Mobile Integrated Health Care		Ambulance		*P* value
	2018-2019	2018-2019	2017-2018	2016-2017	
All response types, No.	1740	1740	1740	1739	
Time on scene, mean (SD), min	55.28 (27.17)	42.18 (15.60)	41.24 (14.13)	40.84 (15.27)	<.001
Time at hospital, mean (SD), min	17.46 (39.71)	41.93 (48.78)	43.14 (51.43)	38.59 (38.54)	<.001
With ED transport, No. (%)	498 (28.6)	1300 (74.7)	1294 (74.4)	1359 (78.1)	
Time on scene, mean (SD), min	60.97 (26.28)	41.08 (14.59)	40.42 (12.53)	40.31 (13.09)	<.001
Time at hospital, mean (SD), min	61.02 (53.44)	56.12 (48.88)	58.01 (51.91)	49.38 (36.98)	<.001
Without ED transport, No. (%)	1242 (71.4)	440 (25.3)	446 (25.6)	380 (21.9)	
Time on scene, mean (SD), min	53.00 (27.20)	45.44 (17.91)	43.62 (17.77)	42.77 (19.72)	<.001

Abbreviation: ED, emergency department.

Overall, the mean (SD) time on scene was 55.28 (27.17) minutes for the MIH team compared with 42.18 (15.60) minutes, 41.24 (14.13) minutes, and 40.84 (15.27) minutes for the matched calls in 2018 to 2019, 2017 to 2018, and 2016 to 2017, respectively. The mean (SD) time at the hospital was 17.46 (39.71) minutes for MIH compared with 41.93 (48.78) minutes, 43.14 (51.43) minutes, and 38.59 (38.54) minutes for ambulance in the 3 matched periods. The mean (SD) total time on task was 72.7 (51.0) minutes for MIH, compared with 84.1 (52.0) minutes, 84.3 (54.1) minutes, and 79.4 (42.0) minutes for matched ambulance in 2018 to 2019, 2017 to 2018, and 2016 to 2017, respectively. The mean time on scene was 10 to 15 minutes longer for MIH compared with regular ambulance, but the MIH team reduced ED transport by 45% to 50%.

### Costs and Mean Cost Per Minute

There were 5 MIH vehicles and 46 ambulances operating during 2018 to 2019, compared with 42 ambulances in 2017 to 2018 and 41 ambulances in 2016 to 2017. During 2018 to 2019, the on-vehicle staff for ambulances included 131 advanced care paramedics and 258 primary care paramedics, compared with 132 and 228, respectively, in 2017 to 2018 and 130 and 207, respectively, in 2016 to 2017. For MIH, the on-vehicle staff included 4 advanced care paramedics, a registered nurse, and an occupational therapist.

As shown in Table 3, the total capital and operating costs for MIH were estimated at $173 645 and $45 214, respectively. The cost for on-vehicle staff was $562 476 and the allocated administration cost was $110 119. The total cost was $891 453 with the total time on task of 126 750 minutes for MIH. The mean cost per minute was estimated at $0.679 for MIH.

**Table 3.** Capital, Operating, and Personnel Costs, and Mean Cost per Minute^a^

Characteristic	Costs, CAD$			
	Mobile Integrated Health Care		Ambulance	
	2018-2019	2018-2019	2017-2018	2016-2017
Capital cost	173 645	3 440 115	3 122 588	3 056 410
Operating cost	45 214	1 022 418	1 075 126	888 305
Personnel cost				
On-vehicle staff	562 476	32 290 254	30 608 375	29 952 734
Administration staff	110 119	3 931 758	3 888 056	4 021 170
Total cost	891 453	40 684 544	38 694 146	37 918 619
Service time, min^b^	126 750	4 525 558	4 712 833	4 112 852
Capital cost per min	0.066	0.142	0.141	0.141
Operating cost per min	0.357	0.226	0.228	0.216
Personnel cost per min	0.256	1.497	1.562	1.575
Mean cost per min	0.679	1.865	1.931	1.933

^a^ All costs were adjusted to 2019 Canadian dollars using the inflation rates published by Bank of Canada (1 CAD$ = $0.7537 US in 2019).

^b^ The service time is the total time on task from responding to the call to final disposition either on scene or at hospital.

The total cost and time on task for regular ambulance responses were $40 684 544 and 4 525 558 minutes for 2018 to 2019, $38 694 146 and 4 712 883 minutes for 2017 to 2018, and $37 918 619 and 4 112 852 minutes for 2016 to 2017. The corresponding mean costs per minute were estimated at $1.865, $1.931, and $1.933 (Table 3).

The costs per 1000 calls for on-scene task, hospital transport, and ED visits for each of the cohorts from the base-case and sensitivity analyses are listed in Table 4. At the base-case analysis, the mean (SD) total cost per 1000 calls associated with MIH was $122 760 ($78 635) compared with ambulance costs of $294 336 ($97 245) for 2018 to 2019, $299 797 ($104 456) for 2017 to 2018, and $297 269 ($81 144) for 2016 to 2017. The sensitivity analyses results are also shown in Table 4.

**Table 4.** Costs of MIH and Ambulance Services per 1000 Calls^a^

Variable	Costs, CAD$			
	MIH		Ambulance	
	2018-2019	2018-2019	2017-2018	2016-2017
Base-case analysis				
On-scene task cost, mean (SD)	37 535 (18 448)	78 666 (29 094)	79 645 (27 289)	78 954 (29 521)
Hospital transport cost, mean (SD)	32 563 (74 059)	78 199 (91 219)	83 315 (99 325)	74 605 (74 508)
Cost of ED visits^b^	52 662	137 471	136 837	143 710
Total cost, mean (SD)	122 760 (78 635)	294 336 (97 245)	299 797 (104 456)	297 269 (81 144)
Sensitivity analyses				
PSM optimal matching				
On-scene task cost, mean (SD)	37 542 (18 448)	78 945 (27 602)	78 197 (27 443)	77 446 (26 950)
Hospital transport cost, mean (SD)	32 507 (74 022)	80 456 (92 098)	88 259 (95 675)	69 288 (65 074)
Cost of ED visits^b^	52 662	137 471	136 837	143 710
Total cost, mean (SD)	122 711 (78 595)	296 873 (97 790)	303 293 (101 019)	290 444 (71 629)
Allocation administrative cost using the number of vehicles				
On-scene task cost	43 505 (21 383)	78 160 (28 907)	79 645 (27 289)	78 954 (29 521)
Hospital transport cost	32 353 (73 582)	77 697 (90 390)	83 315 (99 325)	74 605 (74 508)
Cost of ED visits^b^	52 662	137 471	136 837	143 710
Total cost	128 521 (79 272)	293 328 (96 389)	299 797 (104 457)	297 269 (81 144)
Excluding admin costs				
On-scene task cost	35 191 (17 296)	71 820 (26 562)	72 382 (24 800)	71 345 (26 676)
Hospital transport cost	29 729 (67 614)	71 394 (83 057)	75 717 (90 267)	67 415 (67 328)
Cost of ED visits^b^	52 662	137 471	136 837	143 710
Total cost	117 583 (71 955)	280 686 (88 570)	284 936 (94 931)	282 470 (73 324)

Abbreviations: ED, emergency department; MIH, mobile integrated health care; PSM, propensity score matching.

^a^ All costs were adjusted to 2019 Canadian dollars using the inflation rates published by Bank of Canada (1 CAD$ = $0.7537 US in 2019).

^b^ The cost of ED visits was calculated based on a fixed estimate.

The Figure shows the difference in total cost per 1000 calls between the matched ambulance service and MIH for each of the 3 years. The cost was substantially higher for regular ambulance response than for MIH, with the difference ranging from $163 000 to $180 000 in the base-case and sensitivity analyses.

**Figure.**
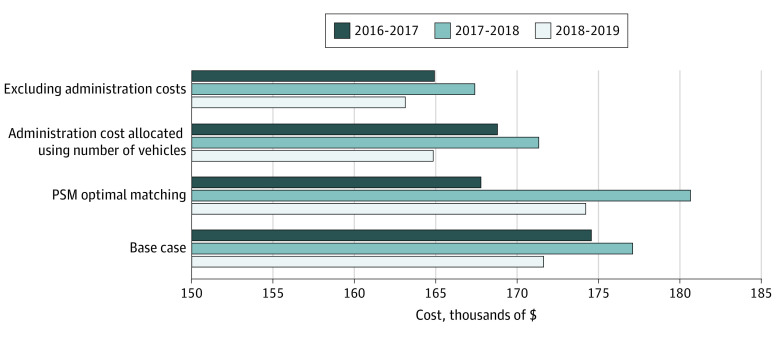
Difference in Cost per 1000 Calls Between Regular Ambulance Response and Mobile Integrated Health Care All costs were adjusted to 2019 Canadian dollars using the inflation rates published by Bank of Canada (1 CAD$ = $0.7537 US in 2019). PSM indicates propensity score matching.

## Discussion

This study estimated and compared the time on task associated with MIH compared with regular ambulances for matched emergency calls identified from the NEMS system. On average, the MIH team spent approximately 10 to 15 minutes longer on scene but reduced ED transport by 45% to 50%, each of which took approximately 40 minutes of ambulance service time. We further converted the time measures to dollar amount using cost per unit time. MIH was associated with savings of approximately 60% of the total cost compared with matched ambulances.

The addition of the MIH teams allows EMS to efficiently respond to low-urgency calls so the ambulance services can be deployed for patients who need stretcher transport or are in more urgent situations. It is important to note that those low-urgency calls were determined according to standard Medical Priority Dispatch System determinants to minimize the likelihood of compromising patient outcomes under this model. This economic analysis shows that a supplemental MIH program using existing EMS infrastructure can provide high value of health care services needed in the community at low additional cost.

Previous evidence shows that an MIH coordination program in the US led to a reduction in ED and hospital admissions, resulting in net savings of $2.4 million over a 6-month period for a Medicare Advantage population.^5^ However, this MIH program was different in that it was led by a physician and consisted of multiple clinical professionals, including emergency medical technician, paramedic, nursing, social worker, pharmacy, and advanced practitioner personnel.^5^ The different team structure, time period, and setting render any direct comparisons with our study impossible, although savings were also demonstrated. Another US study^7^ showed that approximately 15% of all Medicare beneficiaries transported to the ED by EMS were either nonemergent or emergent but primary care treatable, costing approximately $1 billion per year. Our data suggest that low-priority calls could be dealt with by other means, potentially primary care or MIH. This highlights the fact that efficiency measures can certainly be made in the future to result in more savings of scarce resources.

Indeed, a major challenge facing health care systems is resource constraint. EMS call rates have been increasing in Canada. In Ontario alone, between 2012 and 2014, rates of ambulance use increased by 8%, an increase of 17% in costs.^13^ Therefore, it is timely to look for solutions that can counter this increasing EMS demand, but that are efficient in themselves. This study provides real-world Canadian evidence showing that MIH is a promising model that can efficiently meet health care needs in the community. A unique benefit of MIH is avoidance of unnecessary ED transport, which could save a substantial amount of ambulance and hospital resources. Freed-up resources could then be used for urgent cases for which these services are actually needed. For patients, visiting an ED can be tiring and stressful and not always provide the ideal solution to the health care issue. It has been reported that up to half of patients in the ED at any one time experience stress and its effects.^14^ Avoiding an unnecessary ED visit but receiving the appropriate health care solution at home or at the place of the emergency call has the added potential to improve quality of life and satisfaction.

### Strengths and Limitations

There are several strengths in our analysis worth highlighting. First, we used the real-word information routinely collected by EMS. Because of the nature of the EMS services, it is difficult and potentially unethical to design a randomized study to conduct a head-to-head comparison between MIH and ambulance transport. We used propensity score matching to identify a cohort of calls matched on important factors associated with demographic characteristics and the urgency of calls that were received from the same year. We further compared with the matched calls from 2 previous years to adjust for time and other unknown factors that might be associated with the characteristics of calls over time. We also assessed the impact of an alternative propensity score–matching algorithm and costing methods in the sensitivity analyses. We believed that the robustness of our estimates and conclusion have been enhanced by this analytical approach.

This study had some limitations. Although routine quality assurance was a part of this initiative, including qualitative feedback from patients, the EMS database does not record whether those patients called back and required ED transport later. Another limitation is that the cost of ED was an estimated mean cost for an ED visit in Canada and likely underestimated the true cost incurred by patients who were assessed and transported to hospital by EMS. Had all subsequent costs after ED admission been included, the cost savings would likely be even larger in favor of the MIH model. To overcome these limitations, we plan to link the EMS data with a public health administrative database in future studies to allow us to assess outcomes and subsequent use of health care resources, including any increased access to primary care, by those who receive care from MIH. MIH teams provided scheduled follow-up visits between calls. These service times were not recorded in the database. Because the fixed and variable costs associated with MIH have already been included in our analysis, if these between-call services had also been included, the cost per unit time for MIH would decrease, which would further favor the MIH model. It was a challenge when converting time to dollar value because both MIH and ambulances use the same infrastructure and staff support. While recognizing the methodological challenges, we believe that presenting the time in monetary terms can deliver the message in a way that is relevant and understandable by health care budget planners, a target audience of this type of research.

NEMS only started the MIH program in 2018 with 5 response vehicles in operation. It is likely that the MIH team was not able to respond to all calls that met its responding criteria and not all calls serviced by the MIH team represented the best use of this service (eg, those calls that were initially dealt with by the MIH team but had ED transport). There is room for further improvement of this new service model so the resource use can be optimized. To do this, future research should include the collection of detailed information on the health conditions of patients and link EMS data with hospital admission databases. This will help accurately identify and map out the health care needs in the community that can be served by MIH. The MIH team can then be deployed with enhanced precision.

## Conclusions

This economic evaluation’s findings suggest that MIH delivered by NEMS was associated with reduced ED transport and saved substantial savings of EMS staff time and resources compared with ambulance for the matched emergency calls. This service model could be a promising and viable solution to meeting urgent health care needs in the community, while substantially improving the use of scarce health care resources.

## Notes

Supplement. **eTable 1.** Demographic Characteristics of the Emergency Calls Received 2016-2019 **eTable 2.** Emergency Calls Serviced by MIH and Propensity Score Matched Calls Serviced by Ambulance
