# Supplementary material for: Economic Analysis of Mobile Integrated Health Care Delivered by Emergency Medical Services Paramedic Teams
Source: JAMA Netw Open. 2021 Feb 24;4(2):e210055. doi: 10.1001/jamanetworkopen.2021.0055 (PMC7905495; doi:10.1001/jamanetworkopen.2021.0055)
Supplement: Supplement. — eTable 1. Demographic Characteristics of the Emergency Calls Received 2016-2019 eTable 2. Emergency Calls Serviced by MIH and Propensity Score Matched Calls Serviced by Ambulance [file jamanetwopen-e210055-s001.pdf]

Supplemental Online Content

Xie F, Yan J, Agarwal G, Ferron R. Economic analysis of mobile integrated health care delivered by emergency medical services paramedic teams. *JAMA Netw Open*. 2021;4(2):e210055. doi:10.1001/jamanetworkopen.2021.0055

eTable 1. Demographic Characteristics of the Emergency Calls Received 2016-2019
eTable 2. Emergency Calls Serviced by MIH and Propensity Score Matched Calls Serviced by Ambulance

This supplemental material has been provided by the authors to give readers additional information about their work.

© 2021 Xie F et al. *JAMA Network Open.*

eTable 1. Demographic characteristics of the emergency calls received 2016-2019

	2018-19 (N=56,652)	2017-18 (N=55,523)	2016-17 (N=52,536)	p value
**Age, years**				< 0.01
Mean (SD)	59.2 (24.9)	59.7 (24.8)	60.0 (24.8)	
**Gender**				0.04
Female	29,555 (52.2%)	29,270 (52.7%)	27,644 (52.6%)	
Male	26,699 (47.1%)	25,798 (46.5%)	24,497 (46.6%)	
NULL	398 (0.7%)	455 (0.8%)	395 (0.8%)	
**City**				< 0.01
FORT ERIE	3680 (6.5%)	3486 (6.3%)	3071 (5.8%)	
GLANBROOK	6 (0.0%)	4 (0.0%)	5 (0.0%)	
GRIMSBY	2373 (4.2%)	2196 (4.0%)	2255 (4.3%)	
HALDIMAND	26 (0.0%)	21 (0.0%)	17 (0.0%)	
HAMILTON	18 (0.0%)	31 (0.1%)	34 (0.1%)	
LINCOLN	1916 (3.4%)	2070 (3.7%)	1966 (3.7%)	
LONDON	0 (0.0%)	8 (0.0%)	0 (0.0%)	
NIAGARA FALLS	12871 (22.7%)	12654 (22.8%)	12208 (23.2%)	
NIAGARA-O-T-L T	2007 (3.5%)	2037 (3.7%)	1984 (3.8%)	
PELHAM	1347 (2.4%)	1286 (2.3%)	1249 (2.4%)	
PORT COLBORNE	2420 (4.3%)	2430 (4.4%)	2233 (4.5%)	
ST CATHARINES	19973 (35.3%)	19614 (35.3%)	18327 (34.9%)	
STONEY CREEK	76 (0.1%)	76 (0.1%)	95 (0.2%)	
THOROLD	2086 (3.7%)	1996 (3.6%)	1908 (3.6%)	
WAINFLEET	507 (0.9%)	484 (0.9%)	493 (0.9%)	
WELLAND	6485 (11.4%)	6284 (11.3%)	5869 (11.3%)	
WEST LINCOLN	861 (1.5%)	846 (1.5%)	822 (1.6%)	
**Month**				< 0.01
Jan	4,773 (8.4%)	4,838 (8.7%)	4,520 (8.6%)	
Feb	4,238 (7.5%)	4,336 (7.8%)	3,869 (7.4%)	
Mar	4,838 (8.5%)	4,765 (8.6%)	4,340 (8.3%)	
Apr	4,675 (8.3%)	4,471 (8.1%)	4,178 (8.0%)	
May	4,754 (8.4%)	4,825 (8.7%)	4,378 (8.3%)	
Jun	4,743 (8.4%)	4,590 (8.3%)	4,595 (8.7%)	
Jul	5,036 (8.9%)	4,860 (8.8%)	4,642 (8.8%)	
Aug	4,947 (8.7%)	4,622 (8.3%)	4,779 (9.1%)	
Sep	4,669 (8.2%)	4,624 (8.3%)	4,302 (8.2%)	
Oct	4,609 (8.1%)	4,660 (8.4%)	4,314 (8.2%)	
Nov	4,586 (8.1%)	4,368 (7.9%)	3,993 (7.6%)	
Dec	4,784 (8.4%)	4,564 (8.2%)	4,626 (8.8%)	
**Priority Description**				< 0.01
1 (MOH 4)	2,001 (3.5%)	2,178 (3.9%)	1,952 (3.7%)	
2 (MOH 4)	22,430 (39.6%)	22,524 (40.6%)	22,339 (42.5%)	
2T	252 (0.4%)	0 (0.0%)	0 (0.0%)	
3 (MOH 3)	17,743 (31.3%)	16,510 (29.7%)	14,958 (28.5%)	
3T EMERG TRANSFER	1,537 (2.7%)	1701 (3.1%)	1,528 (2.9%)	
4 (MOH 2)	1,187 (2.1%)	843 (1.5%)	962 (1.8%)	
4T SCHEDULED TRANSFER	1,288 (2.3%)	1,272 (2.3%)	861 (1.6%)	
5 (MOH 1)	10,112 (17.8%)	10,398 (18.7%)	9,851 (18.8%)	
5T NON-URGENT TRANSFER	94 (0.2%)	79 (0.1%)	74 (0.1%)	

© 2021 Xie F et al. *JAMA Network Open.*

8 (MOH 8)	8 (0.0%)	18 (0.0%)	11 (0.0%)	

© 2021 Xie F et al. *JAMA Network Open.*

eTable 2: Emergency calls serviced by MIH and propensity score matched calls serviced by ambulance

	MIH		Ambulance		
	**2018-19 (N=1740)**	**2018-19 (N=1740)**	**2017-18 (N=1740)**	**2016-17 (N=1739)**	**p value**
**Age**					0.82
Mean (SD)	57.9 (24.1)	57.3 (25.1)	57.3 (25.6)	58.0 (25.2)	
**Sex**					0.75
Female	896 (51.5%)	870 (50.0%)	892 (51.3%)	918 (52.8%)	
Male	798 (45.9%)	825 (47.4%)	808 (46.4%)	775 (44.6%)	
Not recorded	46 (2.6%)	45 (2.6%)	40 (2.3%)	46 (2.6%)	
**City**					0.78
Fort Erie	47 (2.7%)	43 (2.5%)	49 (2.8%)	48 (2.8%)	
Grimsby	33 (1.9%)	22 (1.3%)	45 (2.6%)	33 (1.9%)	
Haldimand	0 (0.0%)	0 (0.0%)	1 (0.1%)	0 (0.0%)	
Lincoln	33 (1.9%)	28 (1.6%)	25 (1.4%)	39 (2.2%)	
Niagara Falls	366 (21.0%)	380 (21.8%)	393 (22.6%)	381 (21.9%)	
Niagara-On-The-Lake	55 (3.2%)	64 (3.7%)	60 (3.4%)	63 (3.6%)	
Pelham	26 (1.5%)	23 (1.3%)	21 (1.2%)	25 (1.4%)	
Port Colborne	14 (0.8%)	13 (0.7%)	20 (1.1%)	11 (0.6%)	
St Catharines	908 (52.2%)	884 (50.8%)	872 (50.1%)	880 (50.6%)	
Thorold	108 (6.2%)	114 (6.6%)	102 (5.9%)	119 (6.8%)	
Wainfleet	0 (0.0%)	1 (0.1%)	0 (0.0%)	1 (0.1%)	
Welland	144 (8.3%)	160 (9.2%)	147 (8.4%)	136 (7.8%)	
West Lincoln	6 (0.3%)	8 (0.5%)	5 (0.3%)	3 (0.2%)	
**Month**					0.77
Jan	144 (8.3%)	135 (7.8%)	129 (7.4%)	132 (7.6%)	
Feb	155 (8.9%)	155 (8.9%)	127 (7.3%)	172 (9.9%)	
Mar	135 (7.8%)	159 (9.1%)	149 (8.6%)	130 (7.5%)	
Apr	193 (11.1%)	189 (10.9%)	189 (10.9%)	195 (11.2%)	
May	215 (12.4%)	200 (11.5%)	190 (10.9%)	197 (11.3%)	
Jun	220 (12.6%)	215 (12.4%)	239 (13.7%)	216 (12.4%)	
Jul	116 (6.7%)	129 (7.4%)	134 (7.7%)	126 (7.2%)	
Aug	114 (6.6%)	96 (5.5%)	115 (6.6%)	119 (6.8%)	
Sep	76 (4.4%)	70 (4.0%)	81 (4.7%)	82 (4.7%)	
Oct	100 (5.7%)	114 (6.6%)	104 (6.0%)	101 (5.8%)	
Nov	139 (8.0%)	123 (7.1%)	148 (8.5%)	140 (8.1%)	
Dec	133 (7.6%)	155 (8.9%)	135 (7.8%)	129 (7.4%)	
**Priority Description**					0.14
1 (MOH 4)	50 (2.9%)	47 (2.7%)	37 (2.1%)	57 (3.3%)	
2 (MOH 4)	91 (5.2%)	86 (4.9%)	88 (5.1%)	93 (5.3%)	
3 (MOH 3)	775 (44.5%)	813 (46.7%)	785 (45.1%)	781 (44.9%)	
4 (MOH 2)	105 (6.0%)	102 (6.0%)	109 (6.2%)	99 (5.7%)	
5 (MOH 1)	718 (41.3%)	690 (39.7%)	721 (41.4%)	708 (40.7%)	
8 (MOH 8)	1 (0.1%)	0 (0.0%)	0 (0.0%)	1 (0.1%)	

© 2021 Xie F et al. *JAMA Network Open.*
